# Bacterial Colonization of Vagina in Indian Women During Labor and Its Association With Puerperal and Neonatal Sepsis: A Tertiary Hospital Study

**DOI:** 10.7759/cureus.13943

**Published:** 2021-03-17

**Authors:** Sabeena Elliyas, Rajni Gaind, Sandeep Kumar Kanwal, Sarita Singh, Sugandha Arya

**Affiliations:** 1 Department of Obstetrics and Gynaecology, Vardhman Mahavir Medical College and Safdarjung Hospital, New Delhi, IND; 2 Department of Microbiology, Vardhman Mahavir Medical College and Safdarjung Hospital, New Delhi, IND; 3 Department of Pediatrics, Vardhman Mahavir Medical College and Safdarjung Hospital, New Delhi, IND

**Keywords:** puerperal sepsis, neonatal sepsis, vaginal colonization

## Abstract

Objective

The aim was to find the prevalence of colonization of vagina with aerobic bacteria among low-risk Indian women in active labor and its association with early-onset neonatal sepsis (EONS) and puerperal sepsis.

Methods

The study was conducted prospectively from October 2018 to March 2020 in a tertiary hospital in New Delhi, India. Low-risk pregnant women (N=920) in active labor with intact membranes were recruited. High vaginal swabs were collected, cultured by standard methods to detect aerobic bacteria. The primary outcomes were the development of puerperal sepsis and EONS.

Results

In a total of 920 low-risk subjects, vaginal colonization was found in 484 (52.6%), coagulase-negative *Staphylococcus* being the predominant colonizer (13.2%) followed by *Escherichia coli* (8.9%). Multigravida women were at 1.4 times higher risk of colonization than primigravida (odds ratio [OR] 1.399; 95% CI 1.064, 1.84). Women whose sample was collected at the first vaginal examination were at 0.34 times lower risk of colonization as compared to women with more than one vaginal examination (OR 0.34; 95% CI 0.241, 0.481). The incidence of colonization increased with progressive vaginal examinations (p<0.001). None of the colonized women and their neonates developed puerperal sepsis or EONS, respectively.

Conclusion

Vaginal colonization of aerobic bacteria in active labor is not associated with an increased risk of puerperal sepsis or EONS.

## Introduction

World Health Organization reports that more than 500,000 women die every year worldwide due to complications of pregnancy and childbirth [1]. The care during labor is vital for the complete recovery of the woman and her newborn. It is known that sepsis, hemorrhage, and prolonged and obstructed labor are some of the factors at delivery that are responsible for increased maternal and neonatal morbidity [2-3]. Puerperal sepsis is the third most common cause of maternal mortality worldwide [4]. In low- and middle-income countries, puerperal infection is the sixth leading cause of the disease burden in women during their reproductive years and accounts for 15% of total maternal deaths whereas in high-income countries, the death rate due to puerperal sepsis is less than 10% of all causes of maternal death [5]. Puerperal sepsis can be caused by endogenous or exogenous bacterial inoculation of the uterine cavity. Endogenous bacteria are normal commensals in the vagina and rectum but can get carried into the uterus from the vagina by examining fingers or instruments during pelvic examinations particularly following prolonged rupture of membranes, obstructed labor, and traumatic vaginal delivery. Exogenous bacteria can be introduced into the vagina by unclean hands or unsterile instruments. Postpartum puerperal sepsis not only leads to acute morbidity in women but also long-term morbidities like pelvic inflammatory disease and infertility. Postpartum puerperal sepsis was also significantly associated with the occurrence of early neonatal mortality due to early-onset neonatal sepsis (EONS) [6].

EONS presents within 72 hr of birth, and accounts for 10.4% to 85% of total neonatal sepsis [7]. The commonest causative organisms for EONS are endogenous bacteria that are acquired vertically during the process of birth through the mother's reproductive tract. The objective of the study was to find out about maternal colonization during active labor in low-risk women and its possible association with EONS and puerperal sepsis.

## Materials and methods

This was a prospective cross-sectional study conducted from October 2018 to March 2020 in a tertiary-level teaching hospital (Vardhman Mahavir Medical College and Safdarjung Hospital) in New Delhi after approval from the ethical committee of the Institute. With the power of 80%, confidence interval 95%, and lost to follow-up consideration, the sample size derived was 920. It was based on the reported prevalence of vaginal colonization of 62%, EONS in neonates with positive aerobic bacteria culture 7.1%, and EONS with negative bacteria culture 2.9% [8].

All consecutive antenatal women attending the outpatient or emergency department and getting admitted in the labour room were screened to be included in the study. We included low-risk pregnant women with a period of gestation of >28 weeks and in active labor (≥4 cm) with the intact membrane. Women with a history of any foul-smelling vaginal discharge, fever within seven days, antibiotics intake within seven days of admission, infected with HIV and active perineal infection, who had undergone five or more vaginal examinations during labor, undergoing cesarean section (lower segment caesarean section [LSCS]), or having any clinical features suggestive of chorioamnionitis were excluded from the study. Women who gave birth to the neonate with major congenital malformations non-compatible with life and Apgar score <3 at five minutes were also excluded from the study. A total of 10,800 women were screened; 1200 were found eligible and 920 women who consented to be a part of the study were enrolled over the study period.

**Figure 1:**
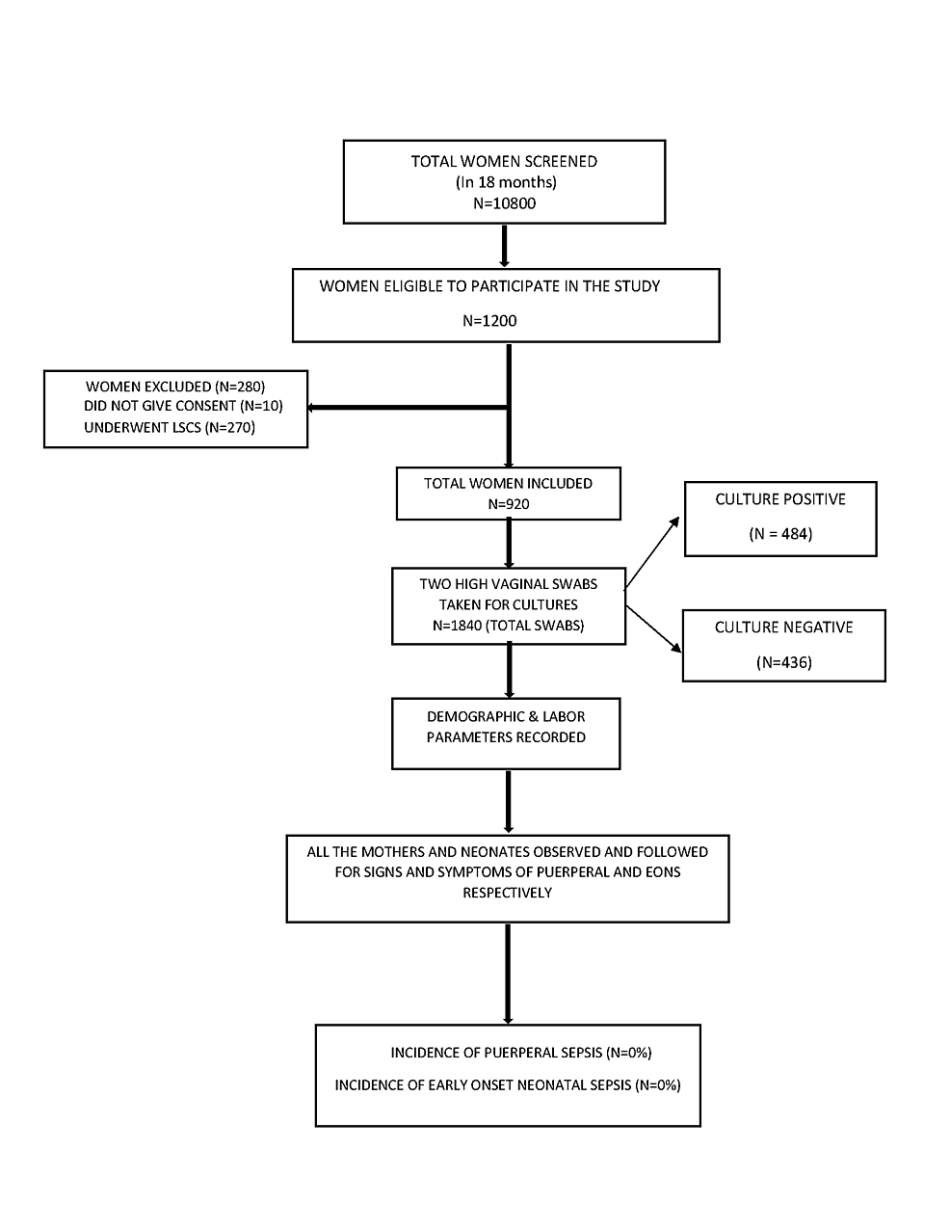
Flowchart showing the study population selection LSCS, lower segment caesarean section

Labor details for the first, second, and third stages of labor were recorded in a predesigned proforma. All of the mothers were observed for the first 72 hr in the hospital for clinical signs and symptoms of sepsis and those with clinical signs were investigated further. They were followed up for two weeks afterward for clinical signs and symptoms of puerperal sepsis. Women were counseled regarding symptoms, for which they might require readmissions such as fever with purulent foul-smelling lochia, pain in the lower abdomen, episiotomy site infection/wound gape, abdominal distention, and other symptoms suggestive of puerperal sepsis (Figure 1).

For collecting samples, the woman was laid in a dorsal position for speculum examination. In one sampling, after asepsis of the perineal area, a clean Cusco’s speculum was inserted into the vagina and two high vaginal swabs (HVSs) were taken from the posterior fornix by a sterile cotton-tipped swab carefully not touching the anal area. The HVSs were transported to the microbiology lab immediately for processing. Out of the two HVSs, one was cultured for six to eight hours on enrichment media followed by subculture on CHROMagar™ StrepB (CHROMagar, Paris, France) for Group B *Streptococcus* (GBS). Another swab was cultured on MacConkey agar and Blood agar. These plates were incubated at 37℃ for 24 hr to allow for the growth of bacterial colonies. The morphology of the colonies grown was studied and one representative of each morphologically distinct colony was picked up and sub-cultured onto new agar plates and was further identified by standard biochemical tests up to species level. Microorganism’s identification was followed by antibiotic sensitivity, performed by the standard procedure according to Clinical and Laboratory Standards Institute (CLSI) 2019 guidelines [9]. Final identification was done by the VITEK® 2 system (bioMérieux, Inc., Durham, NC).

The data was collected in a Microsoft suite and analyzed using SPSS version 24.0 (IBM Corp., Armonk, NY). Following tests of significance were used to evaluate the hypothesis: Pearson chi-square test for proportions, Kolmogorov-Smirnov test for distribution adequacy, unpaired t-test or Mann-Whitney test, or Fisher’s exact test for comparison of means. A p-value of <0.05 was taken as statistically significant. For the significant parameters in vaginal colonization, EONS and puerperal sepsis logistic regression test was used to assess the predictors. Odds ratios (ORs) were used for predicting vaginal colonization.

## Results

The demographic characteristics of participants are given in Table 1.

**Table 1:** Demographic characteristics of enrolled participants pts., participants ^a^Modified Kuppuswamy scale. ^b^Significant value < 0.05.

S. no.	Study participants' characteristics		No. of colonized pts. (N=484) (%)	No. of uncolonized pts. (N=436) (%)	Total pts. (N=920) (%)	p-value^b^
1.	Mother’s age	<20 years	19 (3.9)	19 (4.4)	38 (4.1)	
		20-30 years	398 (82.2)	375 (86)	773 (84)	0.141
		>30 years	67 (13.8)	42 (9.6)	109 (11.8)	
2.	Socio-economic status^a^	Lower	71 (14.7)	61 (14)	132 (14.3)	
		Lower middle	162 (33.5)	134 (30.7)	296 (32.2)	
		Upper lower	246 (50.8)	233 (53.4)	479 (52.1)	
		Upper middle	5 (1)	8 (1.8)	13 (1.4)	
3.	Body mass index (kg/m^2^)	<18.5 (underweight)	62 (12.8)	53 (12.2)	115 (12.5)	
		18.5-22.99 (normal)	314 (64.9)	291 (66.7)	605 (65.8)	
		23-24.99 (overweight)	66 (13.6)	56 (12.8)	122 (13.3)	
		≥25 (obese)	42 (8.7)	36 (8.3)	78 (8.5)	
4.	Parity	Primigravida	202 (41.7)	219 (50.2)	421 (45.8)	0.01
		Multigravida	282 (58.3)	217 (49.8)	499 (54.2)	
5.	Mode of delivery	Spontaneous vaginal	476 (98.3)	426 (97.3)	902 (98)	
		Assisted vaginal	8 (1.7)	10 (2.3)	18 (2)	
6.	Period of gestation (weeks+days)	28-32+6	7 (1.4)	9 (2.1)	16 (1.7)	
		33-36+6	96 (19.8)	40 (15.8)	136 (14.8)	
		≥37	381 (78.7)	387 (88.7)	768 (83.5)	
7.	Birth weight (kg)	≤1.5	2 (0.4)	2 (0.5)	4 (0.4)	
		1.5-2.49	122 (25.2)	91 (20.9)	213 (23.2)	
		≥2.5	360 (74.4)	343 (78.7)	703 (76.4)	
8.	Maturity of delivery	Preterm births (<37 weeks)	106 (21.9)	74 (17)	180 (19.6)	
		Term births (≥37 weeks)	378 (78.1)	362 (83)	740 (80.4)	0.06

The frequency and type of vaginal colonization in women in active labor are given in Table 2.

**Table 2:** Aerobic bacteria isolated in high vaginal swabs CoNS, coagulase-negative *Staphylococcus*; MRSA, methicillin-resistant *Staphylococcus aureus*; MSSA, methicillin-sensitive *S. aureus*

S. no.	Type of organisms	Frequency (N)	Percentage
1	Gram-positive organisms	180	19.6
a.	CoNS	121	13.2
b.	MRSA	27	2.9
c.	MSSA	19	2.1
d.	Enterococcus spp.	11	1.2
e.	Group B *Streptococcus*	2	0.2
2	Gram-negative organisms	121	13.2
a.	Escherichia coli	82	8.9
b.	Klebsiella pneumoniae	18	2
c.	Enterobacter spp.	15	1.6
d.	Pseudomonas aeruginosa	3	0.3
e.	Acinetobacter baumannii	3	0.3
3	A mixture of ≥2 organisms	179	19.4
4	Contaminants	4	0.4
5	Total (colonization)	484	52.6

Out of 920 women, 484 (52.6%) showed vaginal colonization. Vaginal colonization was predominantly by gram-positive organisms in 19.6% followed by a mixture of organisms in 19.4% and gram-negative organisms in 13.2% of enrolled women. The majority of women 121 (13.2%) yielded coagulase-negative *Staphylococcus aureus *(coagulase-negative *Staphylococcus* [CoNS]) followed by *Escherichia coli *in 82 (8.9%), methicillin-resistant *S. aureus* (MRSA) in 27 (2.9%), and methicillin-sensitive *S. aureus* (MSSA) in 19 (2.1%) women. The majority of the women (82.2%) who had vaginal colonization were in the 20-30 years age group.

There was no statistically significant difference observed between vaginal colonization with maternal age, socioeconomic status, body mass index, the period of gestation, term/preterm births, and birth weight of babies, whereas there was a statistically significant relationship between parity and vaginal colonization (p=0.01). The odds predicted that multigravida is 1.4 times more colonized than primigravida with OR 1.399 (95% CI 1.064-1.84).

The 563 samples (61.1%) were collected during a second vaginal examination, 185 samples (20.1%) during the first, 159 (17.2%) during the third, and 13 (1.41%) during the fourth vaginal examination. All mothers for whom samples were collected at the fourth vaginal examination showed vaginal colonization (Table 3).

**Table 3:** Vaginal colonization with number of per vaginal examinations till sample collection CoNS, coagulase-negative *Staphylococcus*; MRSA, methicillin-resistant *Staphylococcus aureus*; MSSA, methicillin-susceptible *S. aureus*

Microorganisms	Number of per vaginal examinations, N (%)			
	1	2	3	4
Acinetobacter	0 (0)	0 (0)	3 (1.9)	0 (0)
CoNS	16 (8.6)	76 (13.5)	28 (17.6)	1 (7.7)
Contaminant	0 (0)	3 (0.5)	1 (0.6)	0 (0)
*Escherichia* *coli*	13 (7)	62 (11)	6 (3.8)	1 (7.7)
Enterobacter spp.	3 (1.6)	6 (1.1)	6 (3.8)	0 (0)
Enterococcus spp.	3 (1.6)	6 (1.10	2 (1.3)	0 (0)
Group B streptococcus	0 (0)	2 (0.4)	0 (0)	0 (0)
Klebsiella pneumoniae	3 (1.6)	6 (1.1)	9 (5.7)	0 (0)
Mixture of 2 organisms	0 (0)	8 (1.4)	6 (3.8)	2 (15.4)
Mixture of >2 organisms	15 (8.1)	77 (13.7)	64 (40.3)	7 (53.8)
MRSA	3 (1.6)	17 (3)	6 (3.8)	1 (7.7)
MSSA	2 (1.1)	13 (2.3)	4 (2.5)	0 (0)
Pseudomonas aeruginosa	0 (0)	2 (0.4)	0 (0)	1 (7.7)
No growth	127 (68.6)	285 (50.6)	24 (15.1)	0 (0)
Total	185 (100)	563 (100)	159 (100)	13 (100)

The multivariate logistic regression predicted that multigravida women were 1.4 times more colonized than primigravida. Women who had undergone sample collection at the first vaginal examination were at 0.34 times lower risk of colonization (Table 4).

**Table 4:** Variables in logistic regression PV exam, per vaginal examination; SE, standard error; OR, odds ratio; df, degrees of freedom

S. no.	Variables	Constant	SE	Wald (chi-square)	df	p-value <0.05	OR (95% CI)
1	BMI <25	-0.03	0.091	0.133	1	0.715	0.96 (0.80-1.15)
2	Lower socioeconomic status	-0.49	0.593	0.690	1	0.41	0.61 (0.17-1.96)
3	Lower middle socioeconomic status	-0.59	0.598	0.973	1	0.32	0.55 (0.17-1.79)
4	Upper middle socioeconomic status	-0.49	0.593	0.69	1	0.40	0.61 (0.19-1.95)
5	Multigravida	0.33	0.140	5.78	1	0.016	1.39 (1.06-1.84)
6	Age of colonization (20-30 years)	0.20	0.396	0.27	1	0.601	1.23 (0.58-2.56)
7	Age of colonization (30-40 years)	0.29	0.219	1.79	1	0.18	1.34 (0.87-2.05)
8	Colonization after the 1st PV examination	-1.07	0.177	37.11	1	0.00	0.34 (0.24-0.48)
9	Colonization after the 2nd PV examination	-10.41	5539.62	0.00	1	0.00	0.00

None of the colonized women and their neonates developed puerperal or early-onset neonatal sepsis, respectively.

## Discussion

In our study, 52.6% of studied women had vaginal colonization with pathogenic aerobic bacteria but none of the colonized women and their neonates developed puerperal or early-onset neonatal sepsis, respectively.

Normally vagina has commensal bacterial flora that undergoes dynamic changes during a woman’s life. The vaginal microbiome is determined by vaginal pH that is less than 4.5 due to the production of lactic acid and availability of glucose for bacterial metabolism. The vaginal flora in prepubescent girls is populated by bifidobacterium due to neutral or alkaline vaginal pH whereas healthy women in the reproductive age group consist mostly of aerobic, lactobacilli that produce lactic acid. The production of lactic acid has indirect effects on pathogens and host defenses. Lactobacilli prevent long-term colonization of various pathogenic bacteria, e.g. *Neisseria gonorrhoeae*, *Escherichia coli*, *Gardnerella vaginalis*, *Peptostreptococcus*, and *Staphylococcus aureus,* not only by producing lactic acid but also by adhering to vaginal epithelial cells. Factors like menstruation, sexual intercourse, antibiotics, stress, and pregnancy cause changes in vaginal microbiology in the reproductive age group [10]. In pregnancy, the vaginal flora gets replaced by polymicrobial organisms, e.g. *G. vaginalis*, anaerobic gram-negative rods such as *Prevotella* species, *Peptostreptococcus* species, *Mycoplasma hominis*, *Ureaplasma urealyticum*, *Staphylococcus*, and often *Enterobacteria* species.

This change in bacterial flora can sometimes lead to asymptomatic or symptomatic bacterial vaginosis and to chorioamnionitis in pregnant women. Furthermore, the consequences are complications in the form of puerperal sepsis, EONS, and preterm labor [11]. In the present study, we focused only on the isolation of aerobic bacteria in vaginal colonization in low-risk asymptomatic pregnant women with intact membranes who presented to the tertiary-level hospital for delivery. The objective was to find out any increased risk of complications like puerperal sepsis and EONS in these subsets of low-risk pregnant women. In the study, CoNS (gram-positive organism) was the most common single isolated organism whereas various studies have reported gram-negative bacteria predominantly [8,12]. The rate of vaginal colonization with maternal age was not found to be different in different age groups (p=0.141). But few studies have reported increased colonization with age, predominantly in >30 years of age [8,12] and some in <20 years of age [13-14]. We did not find any relation between vaginal colonization and BMI (p=0.986) whereas others had found a higher BMI to be a significant risk factor for GBS colonization [15-17].

Multigravidas were at higher risk of colonization than primigravidas. This is similar to studies by Febriani et al., Akkaneesermsaeng et al., and Sharmila et al. [8,14,18]. Multiparous women have had more contact with the health system and may have undergone more genital examinations in their reproductive tenure when compared to the primigravidas. This hypothesis is supported by Top et al., who observed an increase in the occurrence of MRSA and MSSA in multigravidas [19].

As compared to Singaravelu who found the risk of colonization increases in the extremes of socioeconomic status (p<0.005), our study found no relation between socioeconomic class with the rate of colonization (p=0.402) and the type of bacteria [13].

In our study, out of total births in mothers with vaginal bacterial colonization, 78.1% were term and 21.9% were preterm, comparable to others [13]. Vaginal bacterial colonization did not affect the gestational age at the time of labor (p=0.06). Thus, we can suggest that vaginal colonization does not increase the risk of preterm deliveries. Our finding is possibly due to exclusion of subjects with premature rupture of membranes (PROM), preterm premature rupture of membranes (PPROM), and chorioamnionitis that are more likely to predispose to preterm labor.

The incidence of colonization increases with the progressive number of vaginal examinations (p<0.001). In our study, it was observed that with an increasing number of per vaginal examination, colonization with a mixture of two or more organisms increases.

In our study, no cases of EONS were observed irrespective of the vaginal colonization status of the mother. Thus, no association was found between vaginal colonization and EONS. These findings are similar to those of Febriani et al., Buckler et al., and Faro et al. [8,20,21] whereas, Puopolo et al. had reported 30.14 (95% CI 23.92-36.36) times more risk of EONS from mothers with vaginal colonization than without colonization [22].

In the present study, we did not find any association between vaginal colonization detected in active labor with puerperal sepsis also. In our study, the vaginal samples were collected in low-risk women while we excluded women with high-risk factors such PROM, PPROM, chorioamnionitis, maternal fever within seven days before active labor, immunocompromised state such as HIV, and five or more vaginal examinations that are generally the risk factors for puerperal and neonatal infections.

The strengths of our study are that to our knowledge, no such studies on Indian women are available in the common literature, and also a good sample size (N=920). The vaginal samples were taken in active labor when the delivery was imminent, so the culture report can be directly correlated to puerperal and neonatal sepsis. Our study tried to answer whether vaginal colonization in low-risk mothers is a cause of EONS. We studied the type of vaginal colonization with parity and the number of vaginal examinations. The limitation of our study is that it was a single-center study and only aerobic bacteria were looked for; anaerobic and other fastidious bacteria were not cultured. We also did not measure the bacterial load of the cultured bacteria and did not study neonatal colonization.

## Conclusions

Our study concludes that vaginal colonization with potentially pathogenic bacteria in low-risk women in labor usually does not lead to puerperal sepsis or EONS. It reinforces that low-risk mothers should not be given routine antibiotic prophylaxis during labor and postpartum for the prevention of puerperal sepsis and EONS. Multiple vaginal examinations should be avoided during active labor even in a low-risk woman as it increases the risk of vaginal colonization.

Vaginal colonization with pathogenic bacteria might not predispose low-risk women to preterm delivery. The maternal age, BMI, and socioeconomic status of the subjects may not have a significant association with vaginal colonization.
